# Regional Variation of Climatic Influences on West Nile Virus Outbreaks in the United States

**DOI:** 10.4269/ajtmh.14-0239

**Published:** 2014-10-01

**Authors:** Michael C. Wimberly, Aashis Lamsal, Paolla Giacomo, Ting-Wu Chuang

**Affiliations:** Geospatial Sciences Center of Excellence, South Dakota State University, Brookings, South Dakota; Department of Parasitology and Center for International Tropical Medicine, Taipei Medical University, Taipei, Taiwan

## Abstract

The national resurgence of human West Nile virus (WNV) disease in 2012 raised questions about the factors responsible for WNV outbreaks. Interannual climatic variations may influence WNV amplification and transmission to humans through multiple pathways, including mosquito breeding habitats, gonotrophic cycles, extrinsic incubation, avian communities, and human behavior. We examined the influences of temperature and precipitation anomalies on interannual variation in human WNV cases in three regions of the United States. There were consistent positive influences of winter temperatures, weaker and more variable positive effects of spring and summer temperatures, and highly variable precipitation effects that ranged from positive to negative. The overwintering period may be a particularly important climatic constraint on the dynamics of WNV in cold-temperate regions of North America. Geographic differences in the seasonal timing and relative importance of climatic drivers of WNV risk likely reflect underlying variability in key ecological and social characteristics.

## Introduction

West Nile virus (WNV) is a prototypical emerging pathogen that was introduced to the Western Hemisphere in 1999 and has subsequently spread to all 48 conterminous United States and across much of North and South America. WNV has had a substantial public health impact at the national level. In total, 37,088 human WNV cases were reported to the Centers for Disease Control (CDC) through 2012, including 16,196 cases of severe neuroinvasive disease and 1,549 deaths. These effects have been spatially heterogeneous, with geographic clusters of persistently high WNV incidence in the Great Plains, the lower Mississippi Valley, and portions of the Intermountain West.^1^ The incidence of human WNV disease has also varied considerably over time. In the early years of WNV spread, the pattern of invasion was characterized by relatively low incidence of human disease in the first year followed by a large epidemic in the second year and a subsequent decline to endemic levels.^2^ After WNV becomes endemic, human case numbers typically remain lower than during the initial epidemic but are highly variable from year to year.^3^ In particular, the national resurgence of WNV in 2012 after several years of low incidence has raised questions about the factors responsible for triggering WNV outbreaks.^4^

Climatic variation has long been hypothesized to influence the risk of vector-borne and zoonotic diseases through environmental influences on arthropod vectors, avian hosts, microbial pathogens, and human exposure.^5^ Mosquitoes require sufficient rainfall to support habitats for their aquatic stages, and these habitats are further influenced by evapotranspiration and other hydrological processes that control the distribution of water across the land surface.^6^ The length of the gonotrophic cycle of the mosquito vector and the duration of the extrinsic incubation period of the virus both generally decrease with increasing temperature.^7^ As a result, warmer temperatures can lead to larger mosquito populations with a higher proportion of infected mosquitoes. Avian community structure also changes in response to climatic variability, particularly extreme heat and drought.^8^ Because of these myriad influences, climatic anomalies can affect interannual variability in human disease through their impacts on the sizes and spatial distributions of vector and host populations, rates of virus amplification, and risk of virus transmission to humans.

These associations are reflected in previous studies that have documented relationships between climatic variation and the spatial and temporal patterns of human WNV disease. Reisen and others^9^ highlighted associations between positive temperature anomalies and hot spots of WNV activity in the United States from 2002 to 2004. Soverow and others^10^ conducted a national analysis and found that temperature, humidity, and precipitation all had positive effects at lags of 2–4 weeks on the rate of human WNV disease after controlling for seasonality. Chuang and Wimberly^11^ studied human WNV disease in the northern Great Plains and found non-linear but generally positive associations with temperature and greenness anomalies in early spring, moisture anomalies in late spring and early summer, and temperature anomalies during mid to late summer. Chung and others^12^ documented an inverse relationship between the number of days with low temperatures from December to February and the annual rate of West Nile neuroinvasive disease in Dallas County, Texas. Although these studies and others have emphasized the ubiquity of climatic associations with WNV, there is still need for additional research to develop a more generalized understanding of how the many potential climatic drivers affect WNV risk to humans across the United States.

One of the challenges to understanding the climatic triggers of WNV outbreaks is the broad range of environments in which the virus has become endemic. For example, the primary mosquito vectors of WNV vary across diverse regions of the United States, with the different species exhibiting a range of habitat associations, climatic sensitivities, and feeding preferences.^7^ Similarly, geographic variability in bird community composition influences local enzootic cycles and the potential for WNV amplification and transmission to humans. Physical environmental features, including seasonal climate patterns, land cover, topography, and soils, also vary widely across different regions of the United States. As a result, the relationships between climate and vector-borne and zoonotic diseases, such as WNV, are often spatially heterogeneous, with diverse sets of climatic controls operating in different areas. For example, associations between precipitation and WNV incidence varied across the United States, with correlations ranging from positive to negative depending on the geographic area and time period examined.^13^ In the Great Plains of eastern Colorado, human WNV cases were associated with moist spring and dry summer conditions, whereas in the mountainous areas of western Colorado, there were much weaker associations with dry conditions in spring and summer.^14^ Comprehensive and consistent analyses of climatic relationships with WNV risk across multiple regions are needed to better distinguish generalizable relationships from more localized contingencies.

To address this need, the primary of objective of this study was to explore the influences of temperature and precipitation on the interannual variation of human WNV disease in various parts of the United States. To examine these relationships, we used partial least squares (PLS) regression, a multivariate technique that accounts for the correlation structure of multiple climatic variables and provides an intuitive method for visualizing the distribution of human WNV risk in climatic space as biplots. The results showed consistent positive influences of winter temperatures on human WNV risk, weaker and more variable positive effects of spring and summer temperatures, and highly variable precipitation effects that ranged from positive to negative. These findings highlight the potential as well as the limitations of modeling future WNV risk based on temperature and precipitation and suggest future avenues of research to improve our understanding of the complex interactions between climatic variation and WNV.

## Materials and Methods

### Study areas.

**Figure 1.**
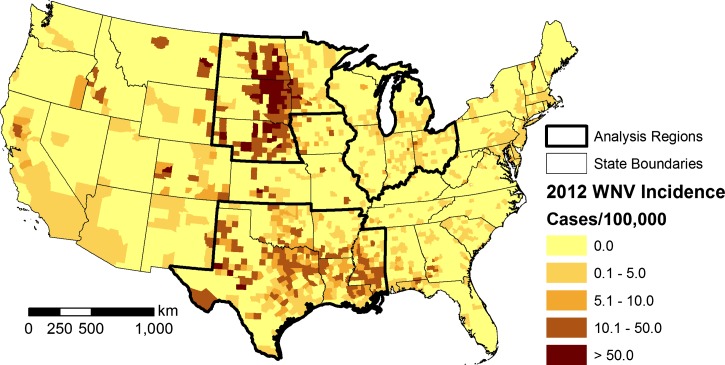
Map of the 2012 county-level incidence of human WNV disease in the United States. Incidence was calculated based on all reported WNV cases, including West Nile neuroinvasive disease and West Nile fever. The three study areas are outlined in bold.

We focused on three regions containing the states that had the highest incidence rates during the resurgence of human WNV cases in 2012 (Figure 1

): the northern Great Plains (NGP), upper Midwest (UM), and southcentral states (SC). The NGP region included Minnesota, Nebraska, North Dakota, and South Dakota. The UM region included Illinois, Indiana, Michigan, Ohio, and Wisconsin. The SC region included Arkansas, Louisiana, Mississippi, Oklahoma, and Texas. These states did not necessarily have the largest numbers of human cases but instead, represented areas where the risk of WNV transmission to humans was assumed to be highest. Altogether, this selection encompassed the 10 states with the highest rates of West Nile neuroinvasive disease in 2012, and all of the selected states had higher than the median rates of West Nile neuroinvasive disease and total human WNV disease (including West Nile fever in addition to West Nile neuroinvasive disease) in 2012 (Table 1).

### Data.

WNV human case data were obtained from the US Geological Survey disease map archive (http://diseasemaps.usgs.gov). The total number of reported human WNV cases per county, including both West Nile fever and West Nile neuroinvasive disease, was used as the dependent variable in the analyses. Human WNV incidence during the initial outbreak years from 1999 to 2003 was likely affected by immunologically naïve avian and human populations and dispersal limitations.^7^ To focus on interannual climatic effects, we analyzed years from 2004 onward, during which time we assumed that WNV was endemic. Positive human cases met the CDC arboviral case definition for neuroinvasive or non-neuroinvasive disease, which included one or more clinical criteria and one or more laboratory criteria.

Although reporting bias of WNV non-neuroinvasive cases is a potential concern for this type of passive surveillance, a strong spatial correlation between total reported WNV cases and neuroinvasive cases was reported in a previous study.^15^ We further explored the temporal associations between annual numbers of neuroinvasive cases and total WNV cases using Spearman rank correlations and Pearson correlations of log-transformed case numbers. We found extremely high correlations (> 0.9) for the majority of states in our study area (Table 1). Thus, we concluded that total numbers of reported WNV cases were sufficient to capture to capture patterns of variability through time similar to those observed in neuroinvasive cases while allowing for a larger sample size.

**Figure 2.**
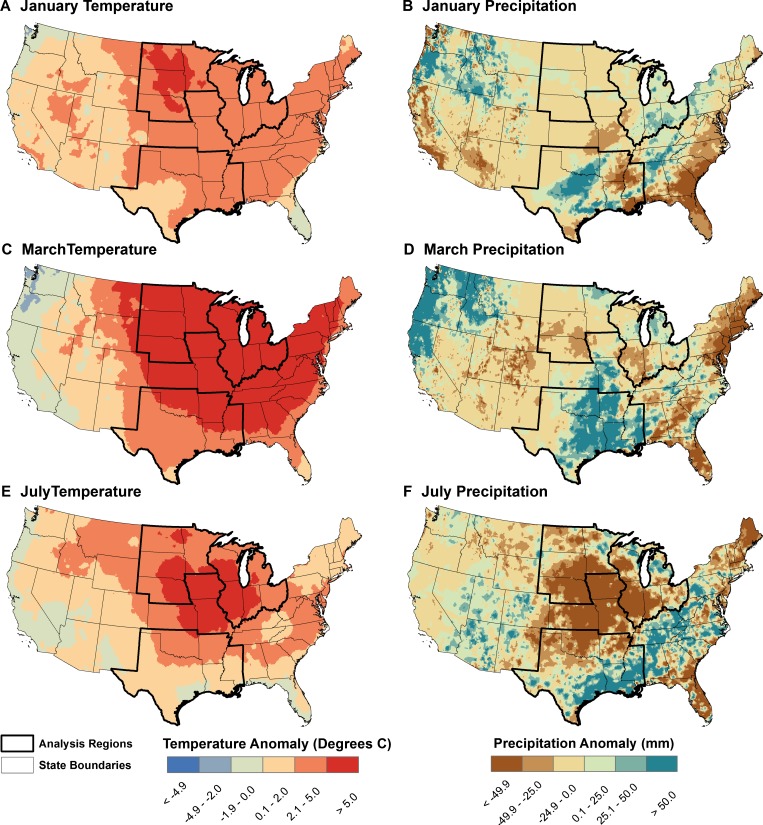
Examples of temperature and precipitation anomalies for 2012 calculated using the NLDAS atmospheric forcing data. Anomalies were computed relative to a 30-year climatology from 1981 to 2010. (**A**) January temperature. (**B**) January precipitation. (**C**) March temperature. (**D**) March precipitation. (**E**) July temperature. (**F**) July precipitation.

Monthly mean temperature and total precipitation from 1981 to 2010 were obtained from the Phase 2 North American Land Data Assimilation System (NLDAS) atmospheric forcing data (Figure 2). These data have a spatial resolution of 0.125° (approximately 10.7 km E-W by 13.9 km N-S at 40° north latitude) and were derived from the assimilation of data from multiple sources, including the North American Regional Reanalysis dataset and the US Climate Prediction Center unified gauge-based precipitation analysis.^16^ Data from one NLDAS grid cell were assigned to each county by overlaying the population-weighted county centroids onto the NLDAS dataset.

### Analysis.

In the analyses, we included only counties with 18 or more cumulative WNV cases (a mean of 2 cases/year) during the 9-year study period (2004–2012) to focus on areas with the highest potential for measuring temporal variability in risk to humans. The study, thus, encompassed 43 counties in the NGP region (*N* = 387), 18 counties in the MW region (*N* = 162), and 57 counties in the SC region (*N* = 513). To quantify the interannual variability of WNV incidence for each county, we calculated the logarithm of the relative rate (LRR) of total WNV cases using the following equation:

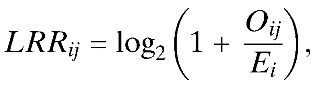


where *i* indexes counties, *j* indexes years, *O_ij_* is the number of observed cases for county *i* and year *j*, and *E_i_* is the expected number of annual cases for county *i* computed as the average number of cases from 2004 to 2012. After taking the binary (base 2) logarithm, *LRR_ij_* = 1 when *O_ij_* = *E_i_*. Values < 1 indicated a lower than expected number of cases, and values > 1 indicated a higher than expected number of cases for a given county.

We analyzed the relationship between county-level annual LRR values and climatic anomalies for the 12 months during and preceding the WNV transmission season. The WNV season in the three study regions occurs mainly between June and September. Therefore, for the LRR in a given year, predictor variables included climate anomalies from January to September of the same year and from October to December of the preceding year. Standardized monthly climatic anomalies from October of 2003 to September of 2012 were computed for temperature and precipitation using the following equation:

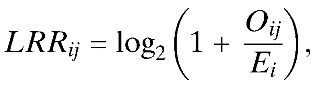


where *k* indexes months, *CLIM_ijk_* is the raw climatic variable (temperature or precipitation) for county *i*, year *j*, and month *k*, *MCLIM_ik_* is the mean, and *SDCLIM_ik_* is the SD of the climatic variable for county *i* and month *k* computed over all 30 years of the climatology (1981–2010).

Because of the potential for strong multicollinearity among the 24 monthly climatic anomaly variables, standard regression techniques are likely to yield inflated parameter variances and unstable parameter estimates. Although variable selection techniques, such as stepwise regression, can be applied to reduce the predictor variables to a smaller subset, these approaches are also sensitive to collinearity and can lead to biases in parameter estimation, inconsistencies in variable selection, and problems associated with multiple hypothesis testing.^17^ We used an alternative approach, PLS regression, as a variable reduction method. PLS regression reduces the original set of predictor variables to a smaller number of latent variables that maximizes the explained variance in the dependent variable.^18^ These latent variables provided a small set of independent climatic effects that was expressed as linear functions of the correlated monthly climate variables.

A separate PLS model was fitted for each of the three study areas. The PLS models were cross-validated by excluding each year (2004–2012) from the training dataset, using the remaining data to fit the PLS model, and comparing the excluded values with predicted values based on climate data for the excluded year. Cross-validation results were used to determine the number of latent factors to include in the final model. PLS regression coefficients were computed for these final models to identify the monthly climatic variables with the strongest effects on LRR. The cross-validation results were used to compute a jackknife estimate of the SE for each regression coefficient.

Biplots of the final models were used to visualize the associations between interannual variability in the rate of human WNV cases and climatic anomalies. The biplots were generated by constructing a scatterplot of the scores of each county/year combination along two latent factors from the PLS model. The relative positioning of points in this plot provided a graphical representation of the climatic similarity of the observations, with close points being more similar and distant points being more different. The size of each point was scaled to reflect the LRR for each county/year combination. Arrows radiating outward from the origin were plotted to illustrate the linear combination of monthly climate variables that comprised each of the latent variables. The distances in the *x* and *y* dimensions from the origin to the tips of the arrows represented the strengths and directions of the associations between the monthly climate variables and the latent variables. To improve the clarity of the biplots and better highlight the most important monthly variables for each latent variable, only variables with a standardized PLS regression coefficient of 0.075 or greater were displayed on the biplots. All analyses were carried out in the R statistical analysis environment.^19^ The sp, raster, and rgdal packages were used for geospatial data processing, and the pls library was used for PLS regression.

## Results

**Figure 3.**
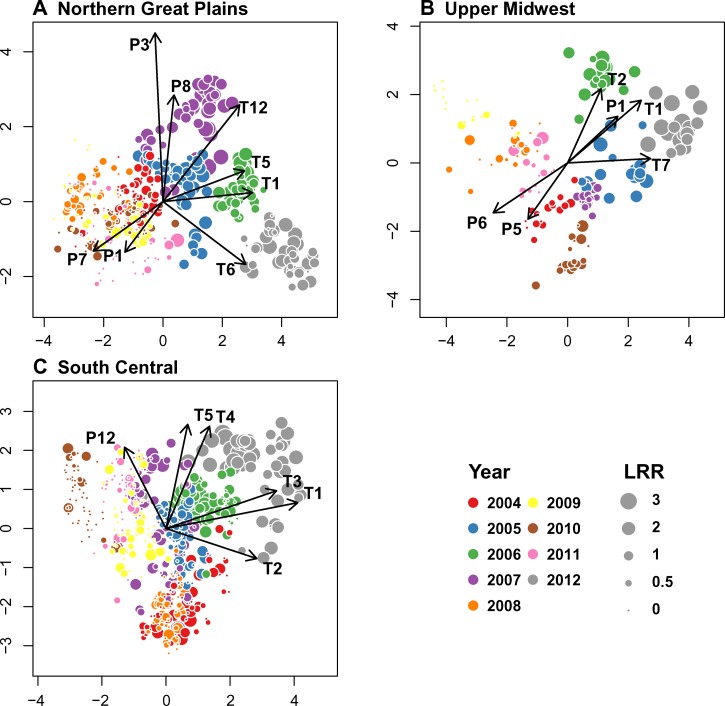
Biplots of PLS regression analysis of the LRR of human WNV disease versus monthly climatic anomalies. The *x* and *y* axes represent latent variables from the PLS models. Circles represent the locations of county/year combinations in climate space, and circle size is proportional to LRR. Arrows represent the relative correlations of climate variables with each PLS axis. (**A**) NGP. (**B**) UM. (**C**) SC. Numbers represent months (1 = January, 2 = February, etc.). P = precipitation; T = temperature.

Biplots provided two-dimensional representations of the distribution of county/year observations in a two-dimensional reduced climate space (Figure 3). Large symbols were clustered in different portions of this climate space than small symbols, indicating that high and low LRR rates were associated with distinctive types of climate anomalies in all three of the regions. The locations of the 2012 observations in these biplots were near the ends of one or both axes, which showed that climatic conditions in 2012 were extreme and distinctive from those observed in other years. In addition, the 2012 observations occurred in the same portions of the biplots as other county/year observations with high LRR values, emphasizing that the LRR values in 2012 were generally consistent with the patterns of climatic associations observed across all counties and years.

In the NGP region, the first two latent factors of the PLS model accounted for 28.9% of the variance in the climatic variables and explained 50.1% of the variance in LRR. County/year observations with high LRR had high scores on axis 1 and were associated with higher-than-average temperatures in December, January, May, and June and lower-than-average precipitation in January and July. High LRR values during the 2007 outbreak had high scores on axis 2 and were associated with higher-than-average temperatures in December and higher-than-average precipitation in March and August.

In the UM region, the first two latent factors of the PLS model accounted for 31.4% of the variance in the climatic variables and explained 64.2% of the variance in LRR. County/year observations with high LRR had high scores on axis 1 and were associated with higher-than-average January and July temperatures and higher-than-average July precipitation. High LRR values, particularly in 2006 and 2012, also had higher scores on axis 2 and were associated with higher-than-average January and February temperatures.

In the SC region, the first two latent factors of the PLS model accounted for 23.4% of the variance in the climatic variables and explained 38.7% of the variance in LRR. County/year observations with high LRR had high scores on axis 1 and were associated with higher-than-average temperatures in January, February, and March. High LRR values also had higher scores on axis 2 and were associated with higher-than-average April and May temperatures and higher-than-average December precipitation.

**Figure 4.**
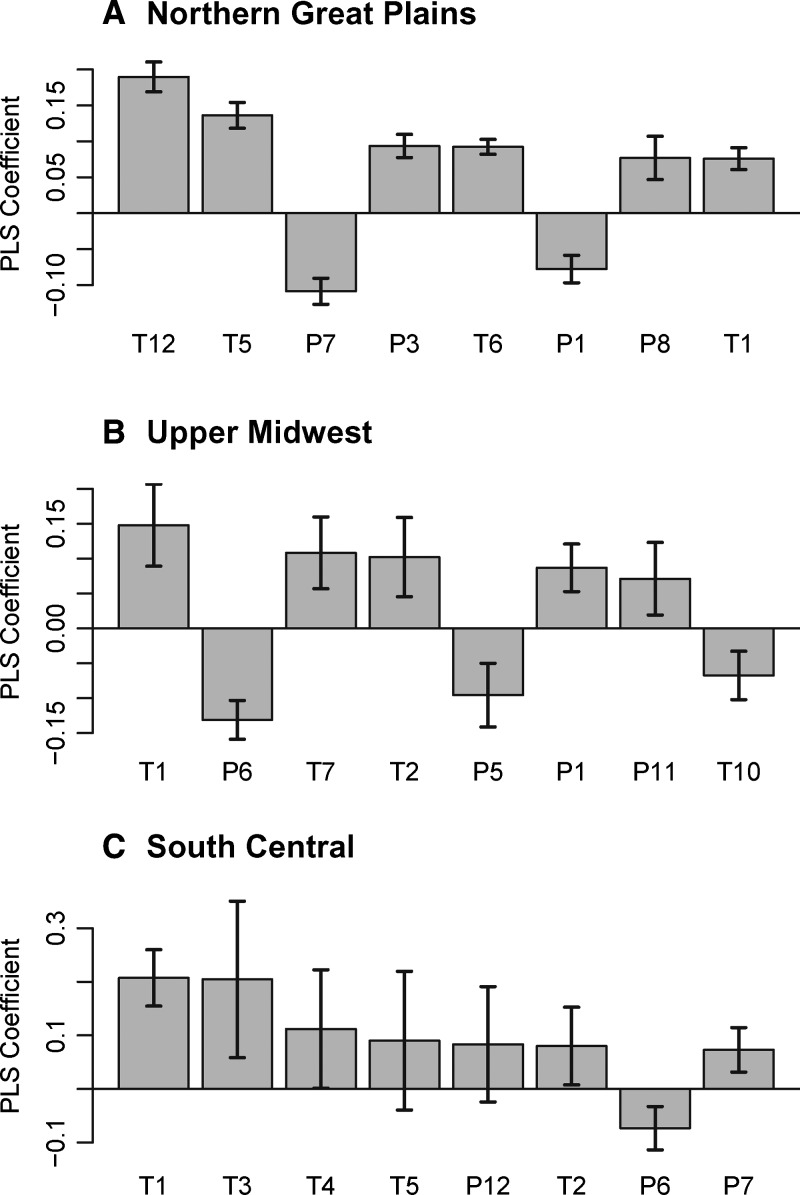
Standardized PLS regression coefficients for the three study regions. The coefficients with the eight highest absolute values are presented in decreasing order for each region. Error bars represent SEs estimated using a jackknife procedure. (**A**) NGP. (**B**) UM. (**C**) SC. Numbers represent months (1 = January, 2 = February, etc.). P = precipitation; T = temperature.

Cross-validation indicated that adding additional axes to the PLS solution beyond the first two increased the coefficient of variation in the model predictions. This result suggested that adding additional latent variables to the PLS solution resulted in overfitting, and therefore, standardized PLS regression coefficients were computed only for the first two latent variables to summarize climatic effects on LRR. The eight variables with the highest standardized PLS coefficients for each region were summarized in Figure 4. Temperature anomalies tended to have the strongest influences in the PLS solutions, and all but one of the temperature anomalies (the October temperature anomaly in the UM region) had positive relationships with LRR. In all three of the study regions, a winter temperature variable (December in the NGP and January in the UM and the SC) had the strongest influence on LRR. Precipitation influences were more variable, with all regions exhibiting a mix of positive and negative coefficients for precipitation. SEs of the standardized PLS regression coefficients were relatively high in the SC for all but the strongest variable (January temperature), indicating greater uncertainty about the climatic influences on WNV than in the other regions.

## Discussion

The strongest and most geographically consistent result from this study was the positive relationship between interannual variability in the rate of human WNV disease and temperature anomalies during the winter months, particularly December and January. This finding suggests that the overwintering phase of the WNV enzootic cycle is a climatically sensitive period that has the potential to influence virus amplification and ultimately, the risk to humans during the subsequent transmission season. However, overwintering is the least understood part of the WNV transmission cycle, and geographic variability in climate as well as mosquito species leads to regional differences in the winter ecology of WNV. Whereas *Culex tarsalis* is the most important WNV vector in the NGP region,^20^,^21^ *Cx. pipiens* is the primary vector in the MW,^22^,^23^ and *Cx. quinquefasciatus* is predominant in the SC region.^24^,^25^ In areas with harsh winters, including most of the NGP and MW regions, small proportions of *Cx. tarsalis* and *Cx. pipiens* can acquire WNV through vertical transmission, enter diapause, and carry the virus through the winter to infect avian hosts the next year.^26^,^27^ In contrast, *Cx. quinquefasciatus* in the SC region does not enter true diapause, can become active during warm periods, and can maintain low levels of horizontal WNV transmission throughout the winter.^28^ It is also possible that the WNV could overwinter in birds and then infect mosquitoes through recrudescent viremias during the next transmission season.^29^ However, it has not yet been shown that birds with long-term infections can successfully transmit the virus to a mosquito.^7^

Obtaining a better understanding of the overwintering period will be critical to furthering our understanding of climatic controls on WNV, a tropical virus that has become widely established in cold-temperate climates in North America.^12^ The positive relationship between winter temperature and WNV risk suggests that mosquito mortality caused by low temperature may reduce the survival of infected mosquitoes and limit the potential for virus amplification and transmission to humans in the following year. Culicine mosquitoes hibernate in a wide variety of natural and anthropogenic habitats, including caves and rock crevices, animal burrows, cellars, and storm drains.^30^ High levels of overwintering mortality of diapausing mosquitoes have been reported, and mortality has been shown to increase during periods with particularly low temperatures and relative humidities.^31^–^33^ Non-diapausing mosquitoes were also shown to have decreased activity during colder weather,^34^ which could reduce the potential for WNV transmission during the winter. Improved knowledge of the specific mechanisms that affect overwintering mosquito activity and survival would help to more precisely identify the types of climatic anomalies that can influence WNV risk during the subsequent summer.

We also found positive relationships with temperature anomalies during the early amplification season from March to May, although the months with the strongest temperature influences were not consistent across regions. These effects are most likely the result of temperature effects on the gonotrophic cycle of the mosquito vector and the extrinsic incubation period of WNV, and they are in agreement with previous studies that have identified positive relationships between temperature and WNV risk.^9^–^11^ Higher temperatures have been shown to increase WNV transmission intensity by reducing the length of the extrinsic incubation period relative to the length of the gonotrophic period (i.e., the time between blood meals), thereby increasing the potential for mosquitoes to acquire and retransmit the virus within their lifetimes.^35^ However, the results from the SC region should be interpreted with caution because of the high SEs associated with the spring temperature coefficients. The weaker fit of the PLS model in this region may indicate that other non-climatic drivers are more important in this relatively warm climate, where temperature is expected to be less of a limiting factor for WNV transmission. Later-season (June to July) temperature anomalies contribute to increased mosquito host-seeking activity that can facilitate continued virus amplification as well as higher rates of infected mosquitoes biting humans.^20^

The geographically variable precipitation associations reflect the results of prior research that have found inconsistent relationships between precipitation and human WNV disease in different years and geographic locations.^13^ These results likely arise from ecological differences among the regions, including variation in the breeding habitat associations of the major vector species.^7^ *Cx. tarsalis* predominantly breeds in rural habitats in the NGP region,^20^ whereas breeding habitats for *Cx. pipiens* in the MW region and *Cx. quinquefasciatus* in the SC region are more closely linked with anthropogenic features, such as underground wastewater systems in urbanized environments.^24^,^36^ Furthermore, differences in hydrological processes that result from distinctive climate, physiography, vegetation, and land use in the various regions influence the flux of moisture throughout the landscape and the consequent distribution of standing water for mosquito breeding. Thus, the inconsistencies in precipitation effects also reflect the limitations of using precipitation as an explanatory variable. Other sources of information, including measurement of standing water and soil moisture from satellite remote sensing,^37^ and predictions of these variables from hydrological models^6^,^14^ offer the potential for measuring surface moisture in a way that is more directly relevant to mosquito biology.

Although most studies of climatic influences on WNV have emphasized the effects of climate variability on mosquito populations, there are other mechanisms through which climate can affect the risk of WNV transmission to humans. For example, avian community composition has been shown to fluctuate from year to year in response to climatic variability.^8^ Different bird species have variable levels of reservoir competence for WNV, and the resulting sensitivity of virus amplification to avian community composition has been well-documented.^38^–^40^ In addition, winter temperature-driven changes in avian reproductive phenology may influence the timing of shifts of mosquito feeding from birds to mammals and thereby, affect the potential for transmission of zoonotic pathogens like WNV to humans.^41^ In combination, this evidence suggests a strong potential for climatic influences on WNV amplification and risk to humans to occur by effects on birds as well as mosquitoes. There is also a growing body of evidence that highlights the sensitivity of human outdoor activities to climatic variation,^42^ suggesting another pathway through which climate may influence exposure to infected mosquitoes and the risk of human WNV disease.

Along with extrinsic factors, such as temperature and precipitation, WNV outbreaks are also sensitive to intrinsic feedbacks within the WNV enzootic system. For example, WNV outbreaks are more likely when the level of herd immunity in avian communities is low, a situation that tends to occur after several seasons of relatively low WNV transmission.^43^ WNV-caused mortality has also had a substantial impact on many bird populations and resulted in persistent declines in many bird species; it has altered avian community structure and likely affected community competence and enzootic transmission of WNV.^44^ Therefore, as with other vector-borne diseases, such as malaria,^45^ it is expected that the dynamics of WNV transmission will ultimately arise through the interactions of these intrinsic and extrinsic factors. Whereas previous research has often focused on lagged effects of climatic variability on vector-borne and zoonotic diseases at scales ranging from a few weeks to a few months, the results from this study support the idea that climatic anomalies during winter and early spring may have longer-term, cascading effects on WNV risk during the subsequent transmission season.^7^ The potential for multiple interacting pathways of climate effects on human WNV risk further emphasizes the potential for spatially heterogeneous climate effects on WNV risk that are contingent on the localized interactions of landscapes, mosquitoes, avian communities, and human behaviors in response to a continually fluctuating climate.

## Figures and Tables

**Table 1:** State-level incidence of human WNV disease in 2012 and interannual correlations of neuroinvasive cases and total WNV cases from 2004 to 2012

Region/state	Neuroinvasive disease		All disease		*r*‡	*ρ*§
	Incidence*	Rank†	Incidence*	Rank†		
UM						
Illinois	1.43	9	2.19	10	0.99	1.00
Indiana	0.69	16	1.15	19	0.97	0.93
Michigan	1.40	10	2.04	12	1.00	0.96
Ohio	0.66	20	1.05	22	0.97	0.92
Wisconsin	0.68	18	0.98	25	0.96	0.98
NGP						
Minnesota	0.63	21	1.30	15	0.98	0.98
Nebraska	2.16	7	10.02	3	0.87	0.75
North Dakota	5.57	2	12.72	2	0.93	0.95
South Dakota	7.44	1	24.36	1	0.97	0.86
SC						
Arkansas	1.49	8	2.14	11	0.98	0.98
Louisiana	3.37	4	7.28	5	0.98	0.93
Mississippi	3.45	3	8.34	4	0.97	0.81
Oklahoma	2.65	6	4.90	7	0.99	1.00
Texas	3.01	5	6.67	6	0.99	1.00

* Per 100,000 population.

† Of all US states in 2012.

‡ Pearson correlation of log-transformed case counts.

§ Spearman rank correlation.
